# Supplementary material for: The causal relationship between the human gut microbiota and pyogenic arthritis: a Mendelian randomization study
Source: Front Cell Infect Microbiol. 2024 Nov 26;14:1452480. doi: 10.3389/fcimb.2024.1452480 (PMC11629706; doi:10.3389/fcimb.2024.1452480)
Supplement: Supplementary file 1 [file DataSheet1.zip › mendelian test/ebi-a-GCST90027604.csv_leave_one_out.pdf]

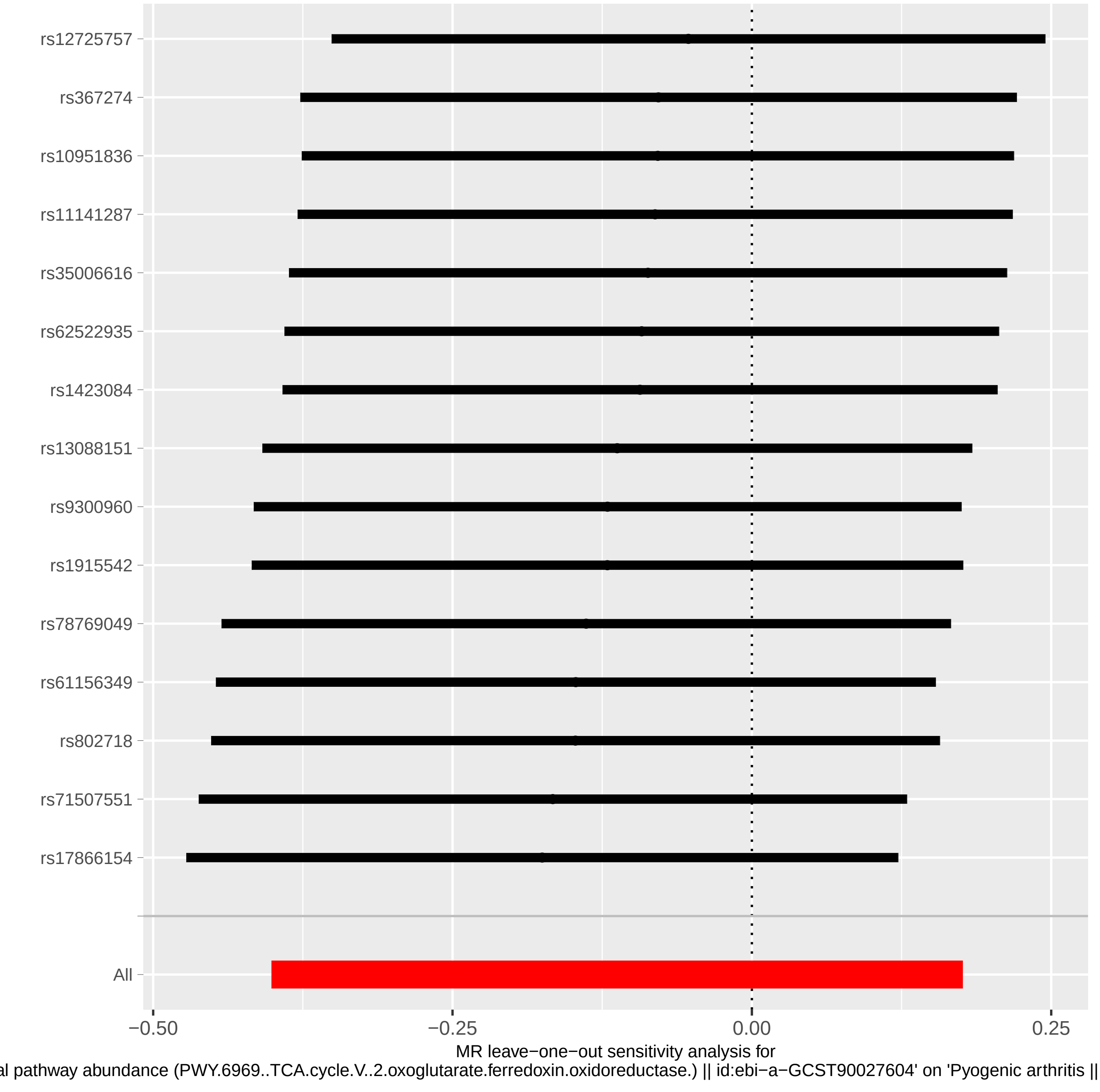

rs12725757
rs367274
rs10951836
rs11141287
rs35006616
rs62522935
rs1423084
rs13088151
rs9300960
rs1915542
rs78769049
rs61156349
rs802718
rs71507551
rs17866154
All
−0.50
−0.25
0.00
0.25
MR leave−one−out sensitivity analysis for
al pathway abundance (PWY.6969..TCA.cycle.V..2.oxoglutarate.ferredoxin.oxidoreductase.) || id:ebi−a−GCST90027604' on 'Pyogenic arthritis ||
